# Use of agro-industrial residue from the canned pineapple industry for polyhydroxybutyrate production by *Cupriavidus necator* strain A-04

**DOI:** 10.1186/s13068-018-1207-8

**Published:** 2018-07-23

**Authors:** Vibhavee Sukruansuwan, Suchada Chanprateep Napathorn

**Affiliations:** Department of Microbiology, Faculty of Science, Chulalongkorn University, Phayathai Road, Patumwan, Bangkok, 10330 Thailand

**Keywords:** Polyhydroxybutyrate, Agro-industrial residue, Canned pineapple industry, Crude aqueous extract, Low-cost PHB production

## Abstract

**Background:**

Pineapple is the third most important tropical fruit produced worldwide, and approximately 24.8 million tons of this fruit are produced annually throughout the world, including in Thailand, which is the fourth largest pineapple producer in the world. Pineapple wastes (peel and core) are generated in a large amount equal to approximately 59.36% based on raw material. In general, the anaerobic digestion of pineapple wastes is associated with a high biochemical oxygen demand and high chemical oxygen demand, and this process generates methane and can cause greenhouse gas emissions if good waste management practices are not enforced. This study aims to fill the research gap by examining the feasibility of pineapple wastes for promoting the high-value-added production of biodegradable polyhydroxybutyrate (PHB) from the available domestic raw materials. The objective of this study was to use agro-industrial residue from the canned pineapple industry for biodegradable PHB production.

**Results:**

The results indicated that pretreatment with an alkaline reagent is not necessary. Pineapple core was sized to − 20/+ 40 mesh particle and then hydrolyzed with 1.5% (v/v) H_2_SO_4_ produced the highest concentration of fermentable sugars, equal to 0.81 g/g dry pineapple core, whereas pineapple core with a + 20 mesh particle size and hydrolyzed with 1.5% (v/v) H_3_PO_4_ yielded the highest concentration of PHB substrates (57.2 ± 1.0 g/L). The production of PHB from core hydrolysate totaled 35.6 ± 0.1% (w/w) PHB content and 5.88 ± 0.25 g/L cell dry weight. The use of crude aqueous extract (CAE) of pineapple waste products (peel and core) as a culture medium was investigated. CAE showed very promising results, producing the highest PHB content of 60.00 ± 0.5% (w/w), a cell dry weight of 13.6 ± 0.2 g/L, a yield ($Y_{{{P \mathord{\left/ {\vphantom {P S}} \right. \kern-0pt} S}}}$) of 0.45 g PHB/g PHB substrate, and a productivity of 0.160 g/(L h).

**Conclusions:**

This study demonstrated the feasibility of utilizing pineapple waste products from the canned pineapple industry as lignocellulosic feedstocks for PHB production. *C. necator* strain A-04 was able to grow on various sugars and tolerate levulinic acid and 5-hydroxymethyl furfural, and a detoxification step was not required prior to the conversion of cellulose hydrolysate to PHB. In addition to acid hydrolysis, CAE was identified as a potential carbon source and offers a novel method for the low-cost production of PHB from a realistic lignocellulosic biomass feedstock.
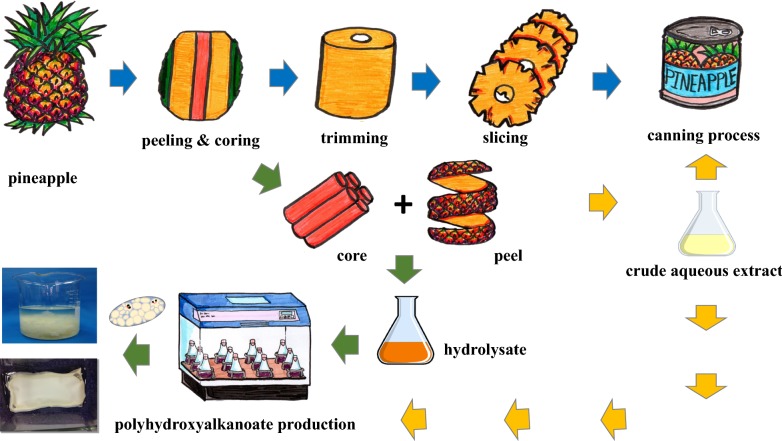

## Background

The current trends and challenges regarding sustainability in industrial biotechnology have stimulated the development of renewable feedstocks that are not sources of food or feed [1]. This transition from simple reducing sugars to alternative renewable raw materials, which have complex structures and require additional procedures, encouraged us to develop simple, economic, and effective processes for the conversion of lignocellulosic biomass, particularly the agro-industrial residue that is abundant in the Southeast Asian region, to fermentable sugars.

Among the various green products that are currently available, bioplastics have received a special attention from academic and industrial researchers in the recent decades. Polyhydroxyalkanoates (PHAs) are microbial polyesters that are synthesized and accumulated in a wide variety of microorganisms as an internal storage energy source [2]. The major drawback to the commercialization of PHAs is their high cost, their precursors, which are mainly high-purity substrates, their long cultivation time, and their extraction and purification process, which is more expensive than those used for the conventional polymers. Even polylactide, PLA, a well-known biodegradable polymer, is synthesized in a hybrid chemical process that is costly to extract and purify. The increasing demand for alternative and renewable raw materials and the use of biodegradable polymers, along with the awareness and promotion of green procurement policies, are motivations expected to benefit the market growth of PHAs.

Lignocellulosic biomass has become an attractive alternative to fossil resources for the production of biofuels and various biochemical reagents, including biodegradable PHAs, which are the most abundant in nature. Cellulosic resources include: agricultural and forest residues, as well as municipal and industrial waste products, which are represented as low-value renewable materials that offer a feasible option for the production of high-value-added products [3]. Several studies have investigated the production of PHAs from cellulose biomass hydrolysate by a variety of microorganisms, including recombinant *Escherichia coli* [4–12]. The direct use of hydrolysates of hemicelluloses as a mixture of sugars for PHA production without the removal of inhibitors has also been explored. For instance, Yu and Stahl found that *Cupriavidus necator* can accumulate to 57% of dry cell weight (DCW) from bagasse hydrolysate in the presence of low inhibitor concentrations [6]. Most recently, Dietrich et al. assessed softwood hemicellulose hydrolysate (mixture of glucose, mannose, galactose, xylose, and arabinose) and the potentially inhibitory lignocellulose degradation products [acetic acid, 5-hydroxymethylfurfural (5-HMF), furfural (FAL), and vanillin] for polyhydroxybutyrate (PHB) production by *Paraburkholderia sacchari* IPT 101, and found that this bacterial strain converted all sugars simultaneously to achieve a maximum PHB concentration of 5.72 g/L and 80.5% (w/w) PHB after 51 h [13]. However, the utilization of hemicellulose hydrolysates for PHB production at an industrial scale necessitates high productivity. In addition, the direct utilization of lignocellulosic waste from the canned pineapple industry as a hemicellulose hydrolysate (as a mixture of sugars) for PHA production has not been investigated.

Pineapple (*Ananas comosus* L. Merr.) is one of the most popular tropical fruits consumed worldwide and an economically significant plant. Approximately 24.8 million tons are produced annually throughout the world (https://www.worldatlas.com/articles/top-pineapple-producing-countries.html). According to the Department of Agriculture, Ministry of Agriculture and Cooperatives, Thailand is ranked first in the production and exportation of pineapple, with an approximately 50% share of the global market [14]. In Thailand, pineapple is exported in various forms to the international market, including canned, raw juice, and various frozen and dried products. In 2016, the quantity of exported canned pineapple was 482,640 tons, which is worth US$615.10 million [15].

**Fig. 1:**
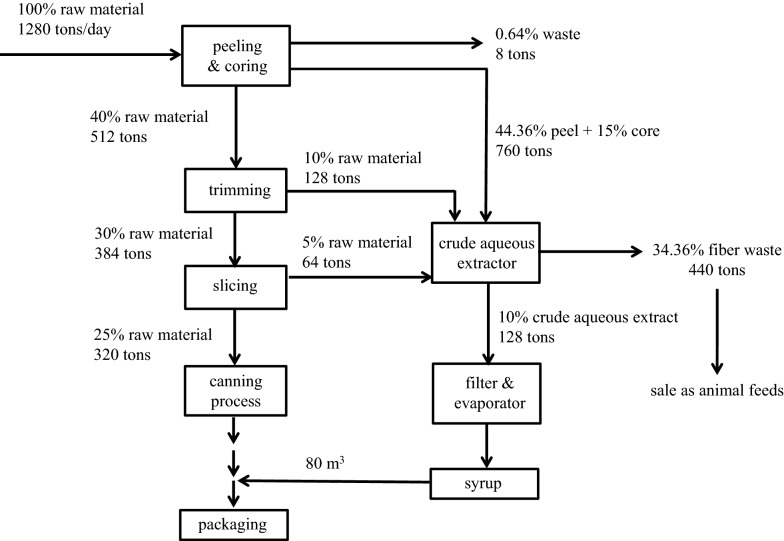
Flowchart of the pineapple waste products generated during industrial pineapple canning

The processing of canned products generates high amounts of agro-industrial waste, and pineapple waste products include 44.36% peel and 15% core with respect to the total raw materials [16] (Fig. 1). Thailand has 75 pineapple processing factories, and these generate approximately 200 tons of agro-industrial waste per day [17]. Pineapple wastes are normally used as animal feed or disposed in landfills, where the wastes might undergo anaerobic digestion, resulting in methane leakage [18]. Leeben et al. analyzed the performance of pineapple processing factories with regard to sustainable development, including waste management [19]. These researchers reported that the pineapple processing factories of Thailand generate large quantities of solid waste and wastewater as well as various amounts of organic content depending on the production capacity, type of technology used, and factory’s size [19]. Modern technology is used in more than 70% of the production lines in large- and medium-sized factories but only in 40–50% of the production process in small factories; therefore, small factories produce higher amounts of waste than large- and medium-sized factories [19]. Large-sized factories manage their peel and core waste products to produce crude aqueous extract (CAE), which is filtered and evaporated to obtain pineapple juice concentrate. The final pulp waste is sold as an animal feed; however, pulp waste is not considered attractive as an animal feed due to its high fiber content and soluble carbohydrates with a low protein content [20]. Small-sized factories might not have a sufficient budget to invest in the production of these by-products [21].

To date, the value-added processing and utilization of pineapple wastes could be a potential source of important compounds, such as sucrose, glucose, fructose, cellulose, fiber, bromelain, phenolics, and cellulose nanocrystals [22, 23]. Researchers in Thailand have focused on the production of fertilizer, improvements in calcareous soil [24, 25], animal feed [26, 27], extraction decomposable pots [17], and plastic reinforcement [28, 29]. The bioeconomy industry has recently become one of the Thai government’s target industries and forms part of the five future industries comprising bioplastics [30]. Thus, this study aimed to fill this research gap by examining the feasibility of using pineapple wastes with the available domestic raw materials for value-added production. To this end, the study focused on the utilization of canned pineapple waste products for PHB production through the development of a rapid, low-cost, and high-yield hydrolysis process.

## Results and discussion

### Compositions of agro-industrial residues

The compositions of pineapple peel, pineapple core, and CAE are presented in Table 1. The major components of pineapple core were found to be 29.5% (w/v) holocellulose [17.2% (w/v) α-cellulose and 12.3% (w/v) hemicellulose] and 1.8% (w/v) lignin. Water content was 89.2% (w/v). Pineapple peel consisted of 36.8% (w/v) holocellulose [22.9% (w/v) α-cellulose and 13.9% (w/v) hemicellulose] and 5.1% (w/v) lignin. Water content was 86.5% (w/v). The sugar composition of CAE was analyzed by HPLC and found to consist of 20.14 g/L sucrose, 24.48 g/L glucose, 2.78 g/L fructose, and 0.30 g/L galactose. The total fermentable sugar concentration in CAE was 47.35 g/L, and this amount included a combined concentration of the PHB substrates glucose and fructose (*S*_PHB_) of 27.26 g/L.

**Table 1:** Characterization of agro-industrial residues

Pineapple wastes	Lignocellulosic biomass compositions (%)					
	α-Cellulose	β-Cellulose	γ-Cellulose	Holocellulose	Lignin	Benzene extractives
Core^a^	17.2	9.55	2.79	29.5	1.82	44.4
Peel^a^	22.9	3.38	10.5	36.8	5.12	29.2

	Sugar content (g/L)				*S*_PHB_ (g/L)	pH
	Sucrose	Glucose	Fructose	Galactose		
Cae^b^	20.14	24.48	2.78	0.30	27.26	2.1

*S*_PHB_ PHB substrates (glucose and fructose)

^a^The chemical compositions of pineapple core and pineapple peel used in this study were determined according to the Technical Association of Pulp and Paper Industry (TAPPI) standard methods

^b^The sugar compositions of CEA were analyzed by HPLC

The lignocellulosic biomass and sugar compositions of CAE reported in this study, however, were somewhat different from those reported previously [31–33]. The biomass and sugar compositions vary depending on the plant age, growth conditions, soil conditions, geographic location, climate, and other environmental factors, such as temperature, stress, and humidity [34].

### Effects of different pretreatment solutions

**Fig. 2:**
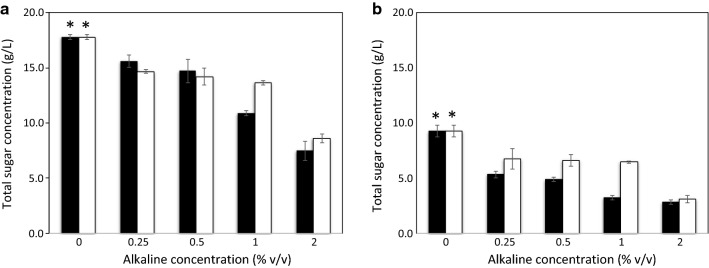
Effects of types and concentrations of alkaline pretreatments on the hydrolysis of **a** pineapple peel and **b** pineapple core. Open bars, Ca_2_OH; solid bars, NaOH. The error bars represent the standard deviations (*n *= 3). An asterisk indicates a significant difference (*P* < 0.05)

Three pretreatment solutions [NaOH, Ca(OH)_2_, and water], with different concentrations of 0.0 (water), 0.25, 0.5, 1.0, and 2.0% (w/v) were systematically investigated in this experiment (Fig. 2). Pineapple peel samples pretreated with water and subjected to 0.5% (v/v) H_2_SO_4_ hydrolysis yielded the highest amount of total reducing sugars (17.8 ± 0.2 g/L) (*P* < 0.05). Interestingly, as shown in Fig. 2a, increasing the NaOH pretreatment solution to 0.25, 0.5, 1, and 2% (w/v) decreased the reducing sugars in PPH to 15.6 ± 0.5, 14.7 ± 0.1, 10.9 ± 0.2, and 7.5 ± 0.8 g/L, respectively. Similarly, the reducing sugars found in PPH decreased to 14.7 ± 0.1, 14.2 ± 0.8, 13.7 ± 0.2, and 8.6 ± 0.4 g/L, respectively, as the concentration of the Ca(OH)_2_ pretreatment solution was increased to 0.25, 0.5, 1, and 2% (w/v). In general, the alkaline pretreatment of lignocellulosic biomass degrades the lignin matrix, resulting in the availability of cellulose and hemicellulose for enzymatic degradation [35]. The effectiveness of this process, however, depends on the lignin content of the lignocellulosic biomass; specifically, the effectiveness obtained with hardwood with a low lignin content is higher than that found with softwood with a high lignin content [36]. As shown in Table 1, the lignin contents in pineapple peel and core were only 5.12 and 1.82% (w/v), respectively. Thus, these materials do not have to be subjected to pretreatment with an alkaline solution. Our results are in accordance with those reported by Jeetah et al., who found that the alkaline pretreatment (2% (w/v) NaOH) of pineapple wastes was not effective in terms of the obtained reducing sugars compared with an un-pretreated sample [37]. In this study, pineapple peel and core are considered softwood with a low lignin content. We proposed that the decrease in reducing sugars observed was a consequence of the extensive hydrolysis of cellulose and sugars by the strongly alkaline solution. It can be concluded that, during alkaline pretreatment, some portions of cellulose and hemicellulose are degraded and removed from the biomass by the action of hydroxide ions [38]. Because alkaline pretreatment steps were found to not be necessary in this study, the total costs of the overall PHA production process can be reduced. The costs of the alkaline solutions Ca(OH)_2_ and NaOH (50% liquid) are ∼ $70 and ∼ $325/ton, respectively. In addition, the absence of an alkaline pretreatment step would reduce the water required for post-pretreatment biomass washing, and subsequently, the waste treatment costs, including capital investment equipment and manufacturing costs, could be reduced [39]. Pineapple peel and core are, therefore, very promising agro-industrial residues for fermentable sugar conversion.

The PPH obtained after water treatment was preliminarily evaluated for PHB production by *C. necator* strain A-04. During a 72-h period, the cell dry weight (CDW) continually increased to 9.68 g/L, and at the end of this incubation, the PHB content reached 17.25% (w/w), indicating that *C. necator* strain A-04 can utilize PPH as a carbon source for growth and PHB production.

### Effect of particle size on the yield of sugars and inhibitors

As shown in Table 2, pineapple core with − 20/+ 40 mesh particle sizes yielded the highest fermentable sugar concentration of 81.0 ± 0.5 g/L; however, these sugars included both PHB substrates (glucose and fructose; 33.6 ± 0.55 g/L) and non-PHB substrates (xylose, arabinose, and galactose; 47.4 ± 0.5 g/L). Pineapple peel with − 20/+ 40 mesh particle sizes also gave the highest xylose concentration, 24.1 ± 0.4 g/L. For both the pineapple peel and core, the + 20 mesh particle sizes yielded the highest PHB substrate concentrations, namely, 43.9 ± 0.4 and 36.9 ± 0.4 g/L, respectively, whereas the − 20/+ 40 mesh particle sizes produced the highest concentrations of xylose, which is not a PHB substrate.

**Table 2:** Effect of particle sizes on the yield of sugars

Pineapple wastes	Particle size (mm)	Sugars (g/L)					Total FS (g/L)	$Y_{{\text{FS/PW}}}$ (g/g)	*S*_PHB_ (g/L)	$Y_{{S_{{\text{PHB}}} /\text{PW}}}$ (g/g)
		Glucose	Fructose	Galactose	Arabinose	Xylose				
1.5% (v/v) H_2_SO_4_										
Core	> 0.841	24.5 ± 0.1	12.4 ± 0.6	8.1 ± 0.6	10.4 ± 0.2	10.3 ± 0.6	65.7 ± 0.4	0.66	36.9 ± 0.4	0.37
	0.841–0.420	31.0 ± 0.1	2.6 ± 0.9	7.6 ± 0.6	8.3 ± 0.6	31.5 ± 0.3	81.0 ± 0.5	0.81	33.6 ± 0.5	0.34
	< 0.420	22.6 ± 0.7	4.0 ± 0.3	8.4 ± 0.4	10.5 ± 0.5	9.1 ± 0.6	54.6 ± 0.5	0.55	26.6 ± 0.5	0.27
Peel	> 0.841	35.8 ± 0.4	7.1 ± 0.4	6.6 ± 0.2	5.3 ± 0.3	13.1 ± 0.9	67.9 ± 0.4	0.68	43.9 ± 0.4	0.44
	0.841–0.420	17.5 ± 0.4	1.5 ± 0.1	5.6 ± 0.1	4.6 ± 0.1	24.1 ± 0.4	53.3 ± 0.2	0.53	19.6 ± 0.3	0.20
	< 0.420	15.3 ± 0.4	1.6 ± 0.1	6.0 ± 0.1	6.6 ± 0.4	15.3 ± 0.4	44.8 ± 0.3	0.45	16.9 ± 0.3	0.17
1.5% (v/v) H_3_PO_4_										
Core	> 0.841	31.4 ± 1.0	25.8 ± 1.1	4.8 ± 0.4	4.1 ± 0.3	3.6 ± 0.4	69.7 ± 0.6	0.70	57.2 ± 1.0	0.57
Peel	> 0.841	20.6 ± 0.8	8.0 ± 0.5	6.2 ± 0.9	8.2 ± 0.3	15.7 ± 0.7	58.7 ± 0.6	0.69	28.6 ± 0.6	0.29

Ten grams of samples for each of three particle sizes were hydrolyzed with 1.5% (v/v) H_2_SO_4_ and 1.5% (v/v) H_3_PO_4_ at 121 °C for 15 min

*S*_*PHB*_ PHB substrates, *FS* fermentable sugars, *PW* pineapple waste

Several reports have stated that reducing the particle size of lignocellulosic biomass improves its digestibility by increasing the total surface area and eliminating mass and heat transfer limitations during hydrolysis reactions [40–43]. However, our results support the findings reported by Harun et al., who found that the hydrolysis of rice straw with ammonia fiber expansion shows reductions in sugar conversion with increases in the size of the milled and cut substrates [44]. The larger cut rice straw particles (5 cm) demonstrated significantly higher sugar conversion than the small particles [44]. Thus, the influence of particle size on biomass digestibility has some limits. We observed that the smallest particles (− 40 mesh, < 0.420 mm) were not fully immersed in the pretreatment solution but rather floated on the surface, which limited their digestibility and resulted in the lowest fermentable sugar and PHB substrate concentrations in all cases tested.

Because the goal of the experiment was to produce PHB by *C. necator* strain A-04, the PHB substrates glucose and fructose were more important than the total fermentable sugars. Therefore, the + 20 mesh particle size, which yielded the highest concentration of PHB substrates, was selected for the next experiment.

### Effects of types and concentrations of acids on the yield of sugars and inhibitors

**Fig. 3:**
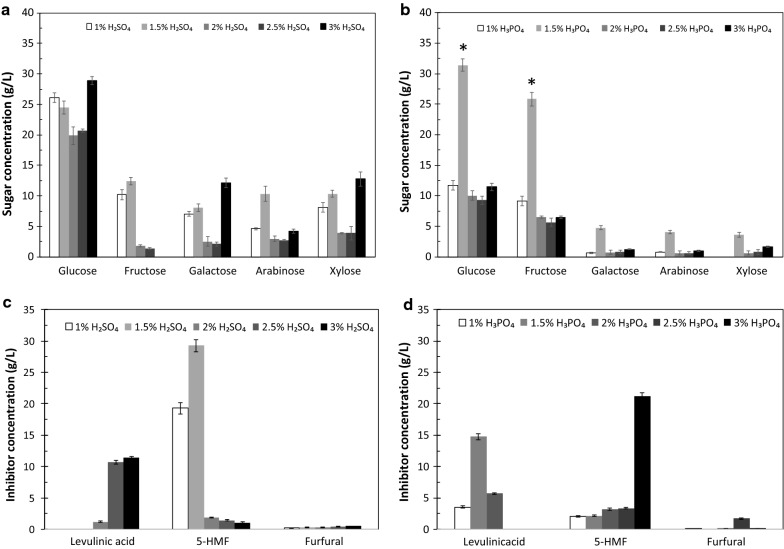
Effects of types and concentrations of acid on **a** the resulting sugar composition after the hydrolysis of pineapple core with 1, 1.5, 2, 2.5, and 3% (v/v) H_2_SO_4_; **b** the resulting sugar composition after the hydrolysis of pineapple core with 1, 1.5, 2, 2.5, and 3% (v/v) H_3_PO_4_; **c** the resulting inhibitor composition after the hydrolysis of pineapple core with 1, 1.5, 2, 2.5, and 3% (v/v) H_2_SO_4_; and **d** the resulting inhibitor composition after the hydrolysis of pineapple core with 1, 1.5, 2, 2.5, and 3% (v/v) H_3_PO_4_. The error bars represent the standard deviations (*n *= 3). An asterisk indicates a significant difference (*P* < 0.05)

**Fig. 4:**
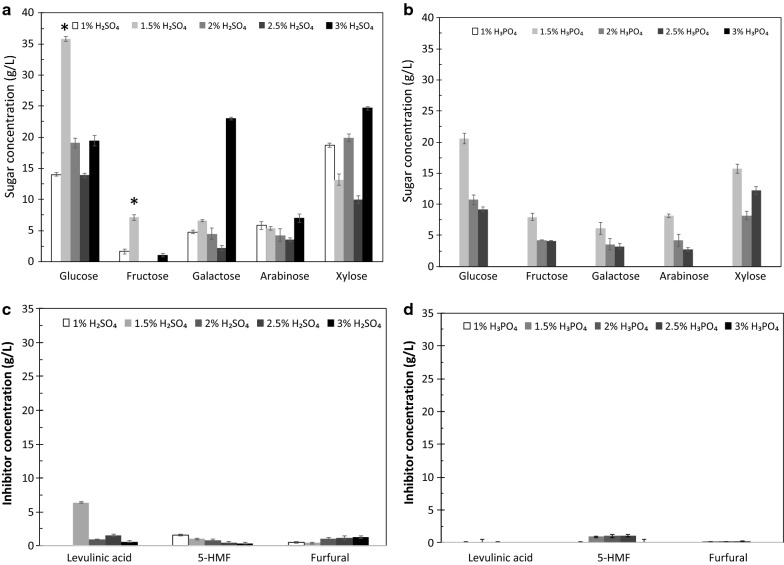
Effects of types and concentrations of acid on **a** the resulting sugar composition after the hydrolysis of pineapple peel with 1, 1.5, 2, 2.5, and 3% (v/v) H_2_SO_4_; **b** the resulting sugar composition after the hydrolysis of pineapple peel with 1, 1.5, 2, 2.5, and 3% (v/v) H_3_PO_4_; **c** the resulting inhibitor composition after the hydrolysis of pineapple peel with 1, 1.5, 2, 2.5, and 3% (v/v) H_2_SO_4_; and **d** the resulting inhibitor composition after the hydrolysis of pineapple peel with 1, 1.5, 2, 2.5, and 3% (v/v) H_3_PO_4_. The error bars represent the standard deviations (*n *= 3). An asterisk indicates a significant difference (*P* < 0.05)

Hydrolysis of pineapple core (Fig. 3) and peel (Fig. 4) with H_2_SO_4_ and H_3_PO_4_ was investigated using concentrations of 1, 1.5, 2, 2.5, and 3% (v/v). Figure 3a shows the sugar composition resulting from the hydrolysis of pineapple core with 1, 1.5, 2, 2.5, and 3% (v/v) H_2_SO_4_, and Fig. 3b shows the sugar composition obtained from the hydrolysis of pineapple core with 1, 1.5, 2, 2.5, and 3% (v/v) H_3_PO_4_. In addition, Fig. 3c illustrates the inhibitor composition resulting from the hydrolysis of pineapple core with 1, 1.5, 2, 2.5, and 3% (v/v) H_2_SO_4_, and Fig. 3d presents the inhibitor composition obtained from the hydrolysis of pineapple core with 1, 1.5, 2, 2.5, and 3% (v/v) H_3_PO_4_. For comparison, Fig. 4a presents the sugar composition obtained by the hydrolysis of pineapple peel with 1, 1.5, 2, 2.5, and 3% (v/v) H_2_SO_4_, whereas the sugar composition obtained after the hydrolysis of pineapple peel with 1, 1.5, 2, 2.5, and 3% (v/v) H_3_PO_4_ is shown in Fig. 4b. Figure 4c reveals the inhibitor composition resulting from the hydrolysis of pineapple peel with 1, 1.5, 2, 2.5, and 3% (v/v) H_2_SO_4_, and Fig. 4d shows the inhibitor composition after the hydrolysis of pineapple peel with 1, 1.5, 2, 2.5, and 3% (v/v) H_3_PO_4_. Glucose was the major product in all the cases. The H_2_SO_4_ hydrolysis of pineapple peel (Fig. 4a) produced PHB substrate levels that were clearly higher than those obtained from pineapple core (Fig. 3a) (*P* < 0.05). This result can be attributed to the higher level of α-cellulose in pineapple peel compared with that in pineapple core. In contrast, the H_2_SO_4_ hydrolysis of the pineapple core (Fig. 3a) produced a xylose level higher than that obtained with pineapple peel (Fig. 4a), consistent with the high hemicellulose content of pineapple core (Table 1). Interestingly, the H_3_PO_4_ hydrolysis of pineapple core (Fig. 3b) yielded a fermentable sugar content of 69.62 ± 0.63 g/L and the highest PHB substrate content, 57.22 ± 1.07 g/L (*P* < 0.05). Thus, the type of acid was found to strongly affect the types of sugar released from the lignocellulosic biomass.

The results of the effects of acid type and concentration on the inhibitors produced are also summarized in Figs. 3c, d and 4c, d. Based on these results, the H_2_SO_4_ (Fig. 3c) and H_3_PO_4_ (Fig. 3d) hydrolysis of pineapple core produced the highest concentrations of inhibitors. Specifically, the highest concentration of 5-HMF, 29.2 g/L, was produced by the 1.5% (v/v) H_2_SO_4_ hydrolysis of pineapple core (Fig. 3c), whereas the hydrolysis of pineapple core with 3.0% (v/v) H_3_PO_4_ produced 21.1 g/L 5-HMF (Fig. 3d). One reason for this result is that pineapple core contains more hemicellulose than pineapple peel (Table 1), and hemicellulose is subsequently hydrolyzed to obtain inhibitors. Therefore, the acid hydrolysis of pineapple peel produced lower amounts of levulinic acid (LA), 5-HMF, and FAL. Xylose and arabinose are known to dehydrate to FAL under acidic conditions, whereas glucose and galactose dehydrate to 5-HMF, which can be further hydrolyzed to LA and formic acid [45]. Acidic conditions thus lead to the formation of FAL and, to a lesser extent, 5-HMF and LA [46].

In this study, we intended to omit the step in which inhibitors are removed from the hydrolysate, because this step reduces the PHB substrate concentration and increases the cost of the large-scale reaction process. Thus, PPH and PCH without detoxification were considered for feasible low-cost PHB production.

### Fermentation of PPH, PCH, and CAE by *C. necator* strain A-04

**Fig. 5:**
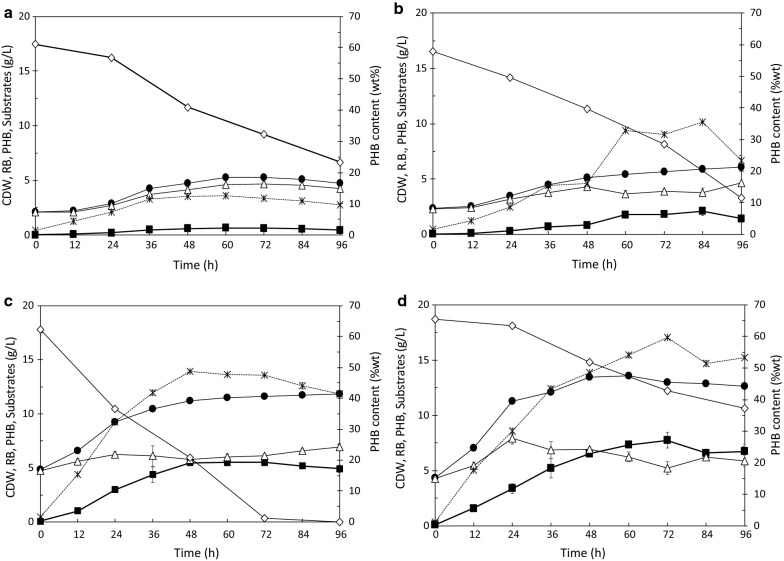
Time course of cell dry weight (g/L), residual biomass (g/L), PHB substrate (g/L), produced PHB (g/L), and PHB content [% (w/w)] during bacterial growth on **a** pineapple peel hydrolysate with a C/N of 200 in production medium, **b** pineapple core hydrolysate with a C/N of 200 in production medium, **c** crude aqueous extract with a C/N of 200 in production medium, and **d** crude aqueous extract without supplementary nitrogen or production medium. The carbon source concentration was 20 g/L in all cases. The error bars represent the standard deviations (*n *= 3)

In this study, PPH produced by 1.5% (v/v) H_2_SO_4_ hydrolysis and PCH produced by 1.5% (v/v) H_3_PO_4_ hydrolysis were selected, because these showed the highest yields of PHB substrates. To evaluate the PHB production performance of *C. necator* strain A-04 using PPH, PCH, and CAE, the concentration of PHB substrates in the production medium was set to 20 g/L. No available nitrogen source was found in PPH, PCH, or CAE; therefore, ammonium sulfate was supplied as a nitrogen source, and the carbon-to-nitrogen (C/N) ratio was set to 200 for shake-flask fermentation [47]. The results are shown in Fig. 5a–d as time courses of the CDW, PHB production, and PHB content obtained with *C. necator* strain A-04. PCH (Fig. 5b) gave a CDW of 5.9 ± 0.2 g/L with a PHB content 35.6 ± 0.1% (w/w) (*Q*_PHB_ = 0.025 g/L/h), whereas PPH (Fig. 5a) resulted in 5.3 ± 0.1 g/L CDW with a PHB content of 12.7 ± 0.6% (w/w) (*Q*_PHB_ = 0.014 g/L/h), even though PPH contained lower amounts of inhibitors than PPC. These results demonstrated that *C. necator* strain A-04 tolerated the various inhibitors present in PPC, and thus, the removal of inhibitors in these mixtures is not necessary prior to their addition to *C. necator* strain A-04.

In addition to PPC and PPH, CAE obtained from pineapple peel and core extracts was also investigated as a carbon source for the growth and PHB production of *C. necator* strain A-04. First, CAE was supplied in the production medium with a C/N ratio of 200 (Fig. 5c). The CDW was enhanced and reached 11.2 ± 0.1 g/L with a PHB content of 48.7 ± 0.2% (w/w) and *Q*_PHB_ = 0.124 g/L/h. This result implied that CAE is more favorable for PHB production by *C. necator* strain A-04 than PPC and PPH. Subsequently, CAE was used as a carbon source alone without any additional nitrogen or medium (Fig. 5d). Under these conditions, the CDW reached 13.6 ± 0.2 g/L, with the highest PHB content of 60.0% (w/w) and *Q*_PHB_ = 0.146 g/L/h. The PHB substrates (glucose and fructose) in CAE with a C/N ratio of 200 in the production medium were consumed more rapidly than those in CAE alone (data not shown). The wild-type *C. necator* strain A-04 efficiently synthesized PHB from PPH [5.3 ± 0.1 g/L CDW with 12.7 ± 0.6% (w/w) PHB], PPC [6.1 ± 0.1 g/L with 35.6 ± 0.1% (w/w) PHB], CAE with N and C/N 200 [11.2 ± 0.1 g/L CDW with 48.7 ± 0.2% (w/w) PHB], and CAE alone [13.6 ± 0.2 g/L with 60 ± 0.4% (w/w) PHB] (Table 4), resulting in high CDW and PHB contents comparable to those obtained with fructose (6.8 g/L CDW with 78% (w/w) PHB) [47]. The obtained results were also comparable to those obtained with *Paracoccus* sp. LL1 grown on corn stover hydrolysate (using the enzyme cellulase) containing 20 g/L sugar, which resulted in 7.18 ± 0.1 g/L CDW with 68.9 ± 1.6% (w/w) PHB [4].

### Kinetic studies of growth, sugar consumption, and PHB production by *C. necator* strain A-04 using PPH, PCH, and CAE

Table 3 summarizes a comparison of the kinetics of cell growth, specific PHB substrate consumption, specific PHB production, yield coefficient of the residual cell mass produced from the consumed PHB substrate, yield coefficient of PHB produced from the consumed PHB substrate, and productivity. The results revealed that CAE without supplementation with nitrogen and medium gave the highest $Y_{{{P \mathord{\left/ {\vphantom {P S}} \right. \kern-0pt} S}}}$, of 0.45 g PHB/g PHB substrate, with a *Q*_PHB_ value of 0.160 g/(L h). PPH produced the highest specific growth rate, 0.010 (1/h), whereas CAE without supplementation with nitrogen and medium produced the lowest specific growth rate, 0.001 (1/h). This effect might be associated to a lack of nutrient elements in the production medium consisting of unsupplemented CAE. PPH also gave the highest value of $Y_{{{X \mathord{\left/ {\vphantom {X S}} \right. \kern-0pt} S}}}$, 0.43 g CDW/g-*S*_PHB_, because PPH contained only trace amounts of inhibitors. In addition, *C. necator* strain A-04 consumed PHB substrates slowly (0.015 g-*S*_PHB_/g-CDW h) when grown on CAE without supplementation with nitrogen and medium, whereas the highest specific consumption rate (0.042 g-*S*_PHB_/g CDW h) was obtained with *C. necator* strain A-04 grown on CAE with a C/N of 200 and production medium, followed by PCH, with a value of 0.032 g-*S*_PHB_/g CDW h. This effect was observed, because PCH contained more fructose than that present in PPH.

**Table 3:** Kinetics of cell growth, sugar consumption, and PHB production by *C. necator* strain A-04 using PPH, PCH, CAE, and CAE without N and medium

Kinetic parameters	PPH	PCH	CAE	CAE
	C/N 200	C/N 200	C/N 200	Without N and medium
PHB substrates (g/L)	20	20	20	20
Maximum PHB concentration (g/L)	0.7 ± 0.1	2.1 ± 0.3	5.5 ± 0.1	7.7 ± 0.5
Maximum cell dry weight (g/L)	5.3 ± 0.1	6.1 ± 0.1	11.2 ± 0.1	13.6 ± 0.2
Maximum PHB content (%wt)	12.7 ± 0.6	35.6 ± 0.1	48.7 ± 0.2	60.0 ± 0.5
Specific growth rate (1/h)	0.010	0.008	0.007	0.001
Specific consumption rate (g-*S*_PHB_/g-CDW/h)	0.019	0.030	0.042	0.015
Specific production rate (g-PHB/g-CDW/h)	0.004	0.007	0.016	0.017
$Y_{{{X \mathord{\left/ {\vphantom {X S}} \right. \kern-0pt} S}}}$ (g-CDW/g-*S*_PHB_)	0.43	0.25	0.09	0.31
$Y_{{{P \mathord{\left/ {\vphantom {P S}} \right. \kern-0pt} S}}}$ (g-PHB/g-*S*_PHB_)	0.10	0.17	0.27	0.45
Productivity (g/(L h)	0.010	0.029	0.091	0.160

*PPH* pineapple peel hydrolysate, *PCH* pineapple core hydrolysate, *CAE* crude aqueous extract

Lignocellulose biomass has mainly been considered a potential substrate for low-cost bioethanol production, and some researchers have studied its potential for PHA production for some time. Selected literature reports describing similar hydrolysis methods were compared with the results of this study, and the data are shown in Table 4. The $Y_{{{P \mathord{\left/ {\vphantom {P S}} \right. \kern-0pt} S}}}$ of 0.17 g PHB/g PHB substrate obtained with PCH was slightly lower than that described in the reported data (0.24 g/g), which was obtained with *C. necator* MTCC-1472 grown on water hyacinth hydrolyzed with H_2_SO_4_ and cellulase as well as activated charcoal for the removal of inhibitors. However, our results demonstrate the ability of *C. necator* strain A-04 to grow on PCH without inhibitor removal. *C. necator* strain A-04 showed superior tolerance to LA; in fact, it tolerated up to 14.7 g/L LA, which was a higher concentration than those previously reported for seven PHA-producing bacteria, namely, *Azohydromonas lata* ATCC 29714, *Bacillus cereus* ATCC 14579, *Bacillus megaterium* ATCC 14581, *Burkholderia cepacia* ATCC 17759, *Pseudomonas oleovorans* ATCC 29347, *Pseudomonas pseudoflava* ATCC 33668, and *Ralstonia eutropha* ATCC 17699 [48]. Furthermore, *C. necator* strain A-04 could utilize inhibitors as carbon sources, because the inhibitor concentrations decreased over time (data not shown).

**Table 4:** Comparison of PHB production from lignocellulosic hydrolysates

Strain	PHA	Carbon source	Hydrolysis	Inhibitors	*T*_max_ (h)	DCW (g/L)	PHB (g/L)	PHB (%wt)	$Q_{{\text{PHB}}}$ (g/(L h)	$Y_{{{P \mathord{\left/ {\vphantom {P S}} \right. \kern-0pt} S}}}$ (g/g)	References
*C. necator* strain A-04	PHB	CAE w/o nitrogen	N	Not remove	48	13.6	7.7	60.0	0.160	0.45	This study
*C. necator* strain A-04	PHB	CAE, C/N 200	N	Not remove	60	11.2	5.5	48.7	0.091	0.27	This study
*C. necator* strain A-04	PHB	PCH	H_3_PO_4_	Not remove	72	6.1	2.1	35.6	0.029	0.17	This study
*C. necator* strain A-04	PHB	PPH	H_2_SO_4_	Not remove	84	5.3	0.7	12.7	0.010	0.10	This study
*C. necator* MTCC-1472	PHB	Water hyacinth	H_2_SO_4_ + cellulase	Activated charcoal	54	12.0	7.0	58.3	0.130	0.24	Radhika and Murugesan [10]
*C. necator*	PHB	Bagasse	H_2_SO_4_	Not remove	48	6.0	3.9	65.0	0.080	ni	Yu and Stahi [6]
*Sphingopyxis*	PHBV	Sawdust	H_2_SO_4_	Not remove	72	0.32	0.23	72.0	0.003	ni	Silva et al. [5]
*Macrogoltabida* LMG 17324											
*Brevundimonas*	PHBV	Sawdust	H_2_SO_4_	Not remove	72	0.25	0.16	72.0	0.003	ni	Silva et al. [5]
*Vesicularis* LMG P-23615											
*Bacillus mycoides*	PHBV	Rice husk	H_2_SO_4_	Not remove	24	1.8	0.39	21.6	0.016	ni	Narayanan et al. [12]

*PPH* pineapple peel hydrolysate, *PCH* pineapple core hydrolysate, *CAE* crude aqueous extract, *ni* not indicate, *PHBV* poly(3-hydroxybutyratae-*co*-3-hydroxyvalerate)

The maximum $Y_{{{P \mathord{\left/ {\vphantom {P S}} \right. \kern-0pt} S}}}$ (0.45 g PHB/g PHB substrate) obtained in this study was higher than the $Y_{{{P \mathord{\left/ {\vphantom {P S}} \right. \kern-0pt} S}}}$ of 0.39 g PHB/g PHB substrate reported by Silva et al. for the utilization of sugarcane bagasse hydrolysate by *Burkholderia cepacia* IPT 101 [5]. Note that CaO and charcoal were used to remove inhibitors [5]. All the above results suggest that pineapple waste products might be a good renewable resource to produce biodegradable polymers and that *C. necator* strain A-04 may be a suitable wild-type bacterial strain for low-cost PHB production from pineapple waste.

## Future research directions

On one hand, the development of lignocellulosic biomass conversion technologies for PHA production has been a research focus over the last decades, and on the other hand, advances in many research areas, such as improved PHA-producing strains, cultivation systems, harvesting technologies, and biocomposite technologies, are required to displace fossil-derived feedstocks by competitive green technologies for PHA production. Our current work aims to establish a technoeconomic platform for PHA production using both wild-type and recombinant strains. The obtained kinetic parameters will be applied to production in a 10-L bioreactor. Furthermore, microcrystalline cellulose has been extracted from pineapple leaves, and its chemical structure has been modified. Finally, biocomposite films of PHB produced from *C. necator* strain A-04 using pineapple waste hydrolysate and pineapple leaf microcrystalline cellulose will be prepared, and their biodegradable, and thermal and mechanical properties will be tested. The outcomes will provide parameters that can be used to guide future research and development.

## Conclusions

The feasibility of using pineapple waste residue from the canned pineapple industry as a lignocellulosic feedstock for PHB production was evaluated. The highest $Y_{{{{S_{{\text{PHB}}} } \mathord{\left/ {\vphantom {{S_{{\text{PHB}}} } {\text{PW}}}} \right. \kern-0pt} {\text{PW}}}}}$ value obtained was 0.57 g/g, and the highest $Y_{{{{\text{FS}} \mathord{\left/ {\vphantom {{\text{FS}} {\text{PW}}}} \right. \kern-0pt} {\text{PW}}}}}$ value was 0.81 g/g. Detoxification was not required prior to the conversion of cellulose hydrolysate to PHB by *C. necator* strain A-04. This bacterial strain showed the ability to tolerate up to 14.7 g/L LA and 2.1 g/L 5-HMF. In addition to acid hydrolysis, CAE was shown to be a potential carbon source and medium, and the CDW, PHA content, and $Y_{{{P \mathord{\left/ {\vphantom {P S}} \right. \kern-0pt} S}}}$ reached 13.6 ± 0.2 g/L, 60 ± 0.4% (w/w) PHB, and 0.45 g PHB/g PHB substrate, respectively. This simple chemical process, which requires neither an alkaline pretreatment nor detoxification steps prior to the PHB production step, could enable the use of crude biomass as the sole carbon source in a scalable biorefinery.

## Methods

### PHB-producing strain

*Cupriavidus necator* strain A-04, a Gram-negative PHB-producing strain isolated from soil in Thailand, was used in this study [47, 49]. The 16S rRNA gene sequence of *C. necator* strain A-04 has been studied and submitted to GenBank under Accession Number EF988626 [47]. Bacterial strain was maintained on a nutrient agar slant at 4 °C by subculturing at monthly intervals. Stock cultures were maintained at − 80 °C in a 15% (v/v) glycerol solution.

### Carbon sources

The agro-industrial residues used in this study were pineapple waste products from the canned pineapple industry, i.e., pineapple peel, core, and CAE. These materials were obtained from Siam Food Products Public Company Limited (a Banbung factory at Tambol Nong-Irun, Amphoe Banbung, Chonburi, Thailand). The pineapple peel and core were dried separately in a hot-air oven (UN55, Memmert GmbH + Co. KG, Schwabach, Germany) at 65 °C for 24 h, milled using a laboratory blender (45,000 rpm, 1800-W, Healthy mix GP 3.5, Taiwan) and then sieved to fractionate the particles into three sizes: less than 0.841 mm (− 20 mesh), 0.841–0.420 mm (− 20/+ 40 mesh), and more than 0.420 mm (+ 40 mesh). The chemical compositions of the cellulose-containing materials were determined according to the Technical Association of Pulp and Paper Industry (TAPPI) standard methods for the following parameters: benzene extractives (TAPPI T 204 cm-07); α-cellulose, β-cellulose, and γ-cellulose (TAPPI T203 om-09); holocellulose (TAPPI T9 m-54; lignin (TAPPI T222 om-15); ash (TAPPI T-211). The composition and concentration of the sugars in CAE were analyzed using a high-performance liquid chromatograph as described in the section detailing the analytical methods.

### Pretreatment and hydrolysis of pineapple waste products from the canned pineapple industry

Ten grams of samples representing each of the three particle sizes + 20 mesh, − 20/+ 40 mesh, and − 40 mesh were pretreated separately using NaOH and Ca(OH)_2_ solutions (0, 0.25, 0.5, 1, and 2% w/v), and followed by autoclaving at 121 °C for 15 min. Water was also run under identical conditions. The pretreated samples were filtered through Whatman filter paper (No. 1, pore size of 11 μm, Sigma-Aldrich Corp., St. Louis, MO, USA), neutralized using tap water, and dried overnight at 80 °C. The neutral-pretreated samples were added to 100 mL of solutions of H_2_SO_4_ and H_3_PO_4_ (1, 1.5, 2. 2.5 and 3% v/v), followed by autoclaving at 121 °C for 15 min [5, 6, 11, 50]. Subsequently, the resulting pretreated samples were filtered, and the filtrate was collected and refiltered through Whatman filter paper. Finally, the pH of the filtrate was adjusted to pH 7.0 using 2 M NaOH to obtain pineapple peel hydrolysate (PPH) or pineapple core hydrolysate (PCH).

### Culture conditions for PHB production from pineapple peel hydrolysate, pineapple core hydrolysate, or crude aqueous extract in production medium

Inocula were prepared in 500-mL Erlenmeyer flasks with 100 mL of preculture medium consisting of 2-g/L yeast extract, 10-g/L polypeptone, and 1 g/L MgSO_4_·7H_2_O, and then grown on a rotary incubator shaker (Innova 4300, New Brunswick Scientific Co., Inc., Edison, NJ, USA) at 30 °C and 200 rpm for 24 h. The cells were harvested by centrifugation and washed to remove any nitrogen source with 0.85% sodium chloride solution. For the synthesis of PHB, the cells were inoculated into a production medium containing mineral salts, consisting of 4.5 g/L Na_2_HPO_4_, 1.5 g/L KH_2_PO_4_, 0.2 g/L MgSO_4_·7H_2_O, 0.05 g/L Fe(III)(NH_4_) citrate (17% Fe), 0.02 g/L CaCl_2_·2H_2_O and 1 mL of trace element solution [0.3 g/L H_3_BO_4_, 0.2 g/L CoCl_2_·6H_2_O, 0.01 g/L ZnSO_4_·7H_2_O, 0.04 g/L MnCl_2_·4H_2_O, 0.03 g/L (NH_4_)_6_Mo_7_O_24_·4H_2_O, 0.02 g/L NiCl_2_·6H_2_O, and 0.01 g/L CuSO_4_·5H_2_O]. The cultivation was performed at 30 °C in 500-mL Erlenmeyer flasks containing 100 mL of production medium with shaking at 200 rpm for 96 h. Culture samples were collected at 12-h intervals. The total fermentable sugar concentrations in PPH, PCH, or CAE were adjusted to 20 g/L, and the carbon-to-nitrogen ratio was set to 200 [51].

### Culture conditions for PHB production from CAE without production medium

Cultivation was performed at 30 °C in 500-mL Erlenmeyer flasks containing 100 mL of CAE without production medium and an adjusted C/N ratio; incubation was conducted on a rotary incubator shaker controlled to 200 rpm for 96 h. Culture samples were taken at 12-h intervals. The sugar contents of CAE, as analyzed by HPLC using the protocol described in the section detailing the analytical methods used in this study, were 24.48 g/L glucose, 20.14 g/L sucrose, 2.73 g/L fructose, and 0.30 g/L galactose. According to the previous reports, these sugars can be used as a carbon source for growth and PHB production by *C. necator* strain A-04 [47].

### Analytical methods

Cell growth was monitored by the CDW, which was determined by filtering 5 mL of the culture broth through pre-weighed cellulose nitrate membrane filters (pore size = 0.22 μm; Sartorius, Goettingen, Germany). The filters were dried at 80 °C for 2 days and stored in vacuum desiccators. The net biomass was defined as the residual biomass, which was calculated by subtracting the amount of PHB from the total biomass. The PHB in dried cells was methyl-esterified using a mixture of chloroform and 3% methanol-sulfuric acid (1:1 v/v) [52]. The resulting monomeric methyl esters were quantified by a gas chromatograph (Model CP3800, Varian Inc., Walnut Creek, CA, USA) using a Carbowax-PEG capillary column (0.25-μm *df*, 0.25-mm ID, 60-m length, Varian Inc.). The internal standard was benzoic acid, and the external standard was PHB (Sigma-Aldrich Corp.). The composition and concentration of the sugars (glucose, fructose, galactose, sucrose, cellobiose, and xylose) in PPH, PCH, and CAE were analyzed using a high-performance liquid chromatograph (Model 626, Alltech Inc., Nicholasville, KY, USA) equipped with an evaporative light-scattering detector (ELSD) (Model 2000ES, Alltech Inc., Nicholasville, KY, USA) and a Rezex RPM monosaccharide column (7.8-mm ID × 300-mm length, Phenomenex Inc., Torrance, CA, USA). Water was used as an elution solvent at a flow rate of 0.6 mL/min. The operating temperature was maintained at 60 °C. The parameters used for ELSD were as follows: the temperature of the drift tube was 105 °C, nitrogen was used as the carrier gas at a flow rate of 2.6 L/min, and the impactor was set in the off position. The composition and concentration of by-products (LA, 5-HMF, and FAL) in PPH and PCH were analyzed using a high-performance liquid chromatograph (model Prostar, Varian Inc., Walnut Creek, CA, USA) equipped with an ultraviolet (UV) detector (wavelength = 285 nm, Prostar 335, Varian Inc., Walnut Creek, CA, USA) and a ChromSpher C18 column (4.6-mm ID × 250-mm length, Varian Inc., Walnut Creek, CA, USA). The mobile phase was methanol:acetic acid:water (12:1:88, v/v) at a flow rate of 1.0 mL/min. The operating temperature was maintained at 25 °C. The total sugar content was determined through a phenol–sulfuric acid assay [53]. The total reducing sugar concentration was determined using a 3,5-dinitrosalicylic acid (DNS) assay [54], and the ${\text{NH}}_{4}^{ + }$ concentration in the culture medium was determined through a colorimetric assay [55].

### Data analysis

All the data presented in this manuscript are representative of the results of three independent experiments and are expressed as the mean values ± standard deviations (SDs). Analysis of variance (one-way ANOVA) followed by Duncan’s test for testing differences among means was conducted using SPSS version 22 (IBM Corp., Armonk, NY, USA). Differences were considered significant at *P *< 0.05.

## List of symbols

### Variables

C/N: molar ratio of carbon to nitrogen (−); $Y_{P/S}$: yield coefficient of PHB produced from consumed PHB substrate (g PHB/g PHB substrate); $Y_{X/S}$: yield coefficient of the residual cell mass produced from the consumed PHB substrate (g RB/g PHB substrate); *T*_max_: time when maximal PHB produced was obtained (h); *Q*_PHB_: PHB productivity (g PHB/L h).

### Abbreviations

PHAs: polyhydroxyalkanoates; PHB: polyhydroxybutyrate; CDW: cell dry weight; RB: residual biomass; LA: levulinic acid; 5-HMF: 5-hydroxymethyl furfural; FAL: furfural; DSC: differential scanning calorimetry; GPC: gel permeation chromatography; PPH: pineapple peel hydrolysate; PCH: pineapple core hydrolysate; CAE: crude aqueous extract.
